# NIPA2 regulates osteoblast function by modulating mitophagy in type 2 diabetes osteoporosis

**DOI:** 10.1038/s41598-020-59743-4

**Published:** 2020-02-20

**Authors:** Wei Zhao, Weilin Zhang, Hongdong Ma, Maowei Yang

**Affiliations:** 1 Department of Orthopedics, the Fourth Hospital of China Medical University, Shenyang, Liaoning China; 2 Department of Orthopedics, the First Hospital of China Medical University, Shenyang, Liaoning China

**Keywords:** Diabetes complications, Type 2 diabetes

## Abstract

The highly selective magnesium transporter non-imprinted in Prader-Willi/Angelman syndrome region protein 2 (NIPA2) has recently been associated with the development and progression of type 2 diabetes osteoporosis, but the mechanisms involved are still poorly understood. Because mitophagy is involved in the pathology of type 2 diabetes osteoporosis, the present study aimed to explore the relationship among NIPA2, mitophagy and osteoblast osteogenic capacity. NIPA2 expression was reduced in C57BKS background db/db mice and *in vitro* models of type 2 diabetes osteoporosis, and the activation of mitophagy in primary culture osteoblast-derived from db/db mice and in high glucose-treated human fetal osteoblastic cells (hFOB1.19) was observed. Knockdown, overexpression of NIPA2 and pharmacological inhibition of peroxisome proliferator-activated receptor γ coactivator 1-α (PGC-1α) showed that NIPA2 increased osteoblast function, which was likely regulated by PTEN induced kinase 1 (PINK1)/E3 ubiquitin ligase PARK2 (Parkin)-mediated mitophagy via the PGC-1α/forkhead box O3a(FoxO3a)/mitochondrial membrane potential (MMP) pathway. Furthermore, the negative effect of mitophagy on osteoblast function was confirmed by pharmacological regulation of mitophagy and knockdown of Parkin. Taken together, these results suggest that NIPA2 positively regulates the osteogenic capacity of osteoblasts via the mitophagy pathway in type 2 diabetes.

## Introduction

The prevalence of type 2 diabetes mellitus (T_2_DM) increases with economic development and aging, exerting a substantial economic burden on the healthcare system^1^. Loss of bone mass is a well-known risk factor associated with osteoporotic fractures in T_2_DM patients, leading to significant impairment in their daily functions and quality of life^2^. Although large studies have been carried out in the past decade to study the possible pathological mechanism of type 2 diabetes osteoporosis, the main active pathological mechanism is complicated and remains controversial. Recent studies have widely reported that the oxidative stress mediated by divalent metal ions plays a key role in the pathogenesis of osteoporosis^3–5^, and we previously confirmed the critical effect of divalent metal ions on type 2 diabetes osteoporosis^6,7^.

As a divalent metal ion, magnesium is the second most abundant intracellular cation after potassium, and approximately 50–60% of the total magnesium in the body is stored in bones^8^. Additionally, while previous studies have shown the relationship between intracellular magnesium deficiency and T_2_DM^9,10^, its role in type 2 diabetes osteoporosis remains unclear. Mg^2+^ influx and efflux in mammalian cells are mediated by metal ion transporters. NIPA2 was found the only transporter that is highly selective for Mg^2+^ and helps maintain Mg^2+^ influx^11^. Furthermore, an epidemiological study showed a genetic variation of NIPA2 in T_2_DM^12^, but few studies have provided experimental evidence of the relationship between NIPA2 and type 2 diabetes osteoporosis, and the specific mechanism is still unclear. Therefore, our study was performed to determine the effect of intracellular magnesium deficiency induced by NIPA2 deletion on type 2 diabetes osteoporosis and to investigate the possible pathological mechanism.

The involvement of autophagy in the pathological mechanism underlying type 2 diabetes osteoporosis has been widely investigated in recent studies, but the results remain controversial^13–16^. These different conclusions may be due to the combined effect of autophagy on specific organelles. Because mitochondria are the key sites of glucose metabolism, we speculate that a precise investigation of mitophagy will shed light on the role of autophagy in type 2 diabetes osteoporosis. Sheng *et al*. reported the positive effect of mitophagy on hyperglycemia-mediated mitochondrial damage in diabetic nephropathy^17^. In contrast, Wang *et al*. found that attenuation of mitophagy may reduce mitochondrial loss and increase mitochondrial biogenesis in the skeletal muscles of diabetic rats^18^, but the effect of mitophagy on type 2 diabetes osteoporosis remains unclear. In this study, we aimed to examine the regulatory effect of intracellular magnesium on mitophagy in type 2 diabetes osteoporosis and the specific mechanism by which this effect is exerted.

Although magnesium and mitophagy play critical roles in the process of osteoporosis, the interaction of magnesium transporter NIPA2 and mitophagy in type 2 diabetes osteoporosis is not known. We found that the absence of NIPA2 in osteoblasts leads to deficiency of intracellular magnesium under type 2 diabetes environment. Magnesium was reported tightly linked to mitochondria physiology. Liu *et al*. found that magnesium may improve mitochondrial function by increasing ATP and decreasing mitochondrial ROS and calcium overload^19^. Ha *et al*. reported the regulation of magnesium on mitochondrial biogenesis via AMPK signals pathway^20^. Chen *et al*. found that magnesium may increase the mitochondrial membrane potential of bone marrow mesenchymal stem cells, which is one of the key factors of inducing mitophagy^21^. But few researches showed the relationship between NIPA2 and mitophagy in type 2 diabetes osteoporosis. Therefore, we aimed to investigate the specific mechanism of NIPA2 regulating mitophagy and osteogenic capability of osteoblasts.

We speculated that deletion of NIPA2 leads to a deficiency in intracellular magnesium, which may affect osteoblast function by modulating mitophagy in type 2 diabetes osteoporosis. Therefore, the expression of NIPA2 in a widely used T_2_DM model, db/db mice and *in vitro* models of type 2 diabetes osteoporosis were examined, and the level of mitophagy in high glucose (HG)-treated hFOB1.19 osteoblasts was analyzed. After knockdown and overexpression of NIPA2, its effects and possible mechanisms on mitophagy and osteoblast function were evaluated. The effects of mitophagy on osteoblast function were also observed via the pharmacological regulation of mitophagy and knockdown of Parkin, the key mitophagy gene. This research aimed to further our understanding of the possible pathological mechanisms underlying type 2 diabetes osteoporosis by investigating the potential interaction among NIPA2, mitophagy and osteoblast function to develop potential target treatments.

## Results

### Bone microstructure of type 2 diabetes osteoporosis mice

**Figure 1:**
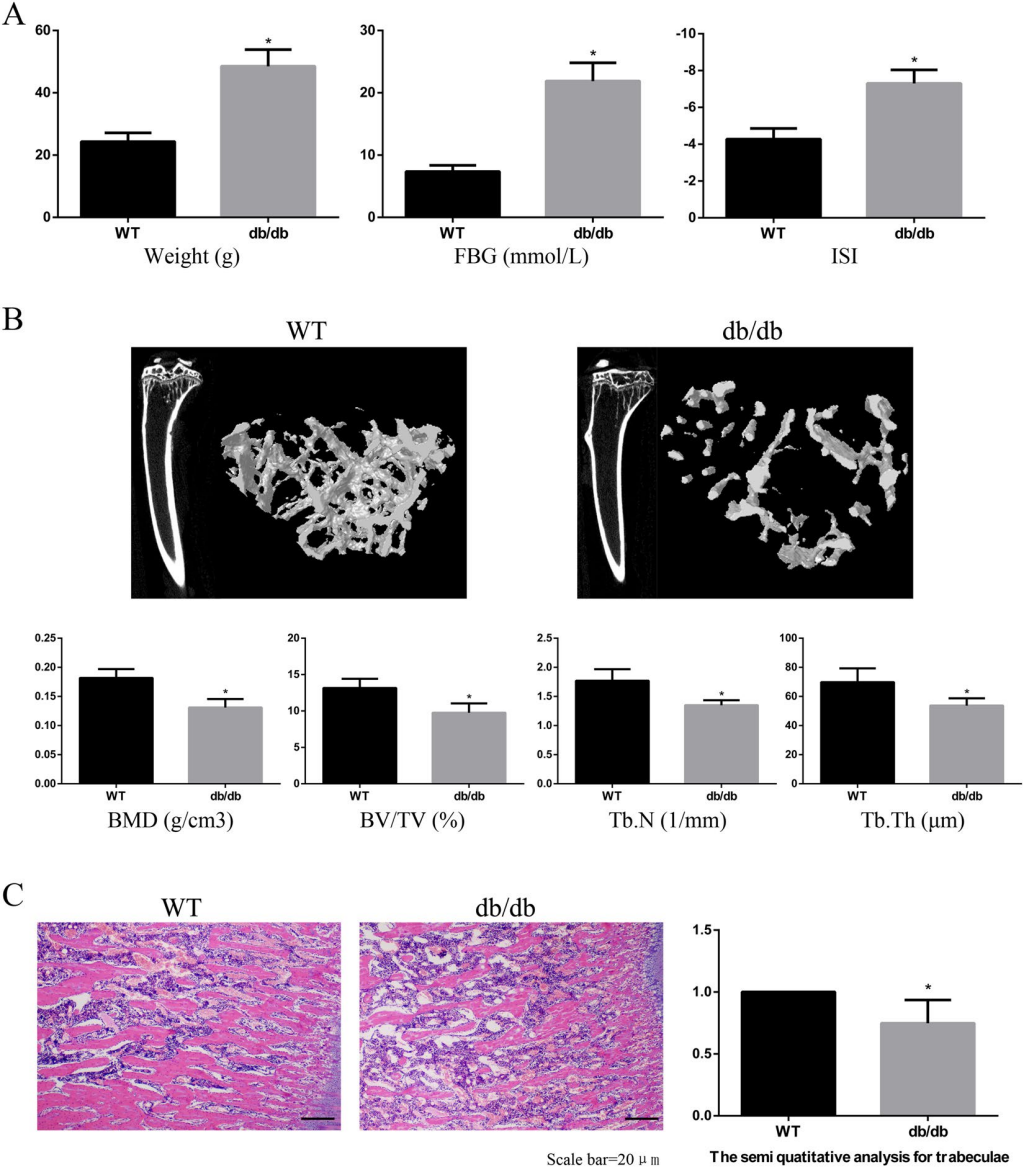
Bone microstructure of type 2 diabetes osteoporosis mice. C57BKS db/db mice were included in the db/db group (n = 15), and their lean littermates C57BKS mice were included in the WT group (n = 15). **(A)** The body weight and FBG levels in the db/db group were significantly higher than those in the WT group at 12 weeks of age, while the ISI levels were consistently lower in the db/db group than in the WT group. **(B)** Micro-CT scanning at 12 weeks. The BMD, BV/TV, Tb.N and Tb. Th values in the db/db group were significantly lower than those in the WT group. **(C)** HE staining at 12 weeks. The number and thickness of trabecular bone were significantly lower in the db/db group than those in the WT group. HE staining data are expressed as the fold induction relative to the control. Values are presented as the mean ± SD. *P < 0.05 vs. WT, n = 15 per group.

In this study, we utilized db/db mice, a widely used mouse model of T_2_DM combined with osteoporosis^22^. At 12 weeks of age, the body weight and blood glucose values of the db/db group were significantly higher than those of the WT group, and the ISI values in the db/db group were significantly lower than those in the WT group (Fig. 1A). Then, we assessed the indexes of bone microstructure, including BMD, BV/TV, Tb.N, and Tb.Th, using micro-CT scanning (Fig. 1B). The bone microstructure of the right tibia was significantly worse in the db/db group than in the WT group. The results of HE staining confirmed these findings (Fig. 1C). These results validated our animal model of type 2 diabetes osteoporosis.

### ***In vivo*** downregulation of NIPA2 in the bone tissues and osteoblasts of type 2 diabetes osteoporosis mice

**Figure 2:**
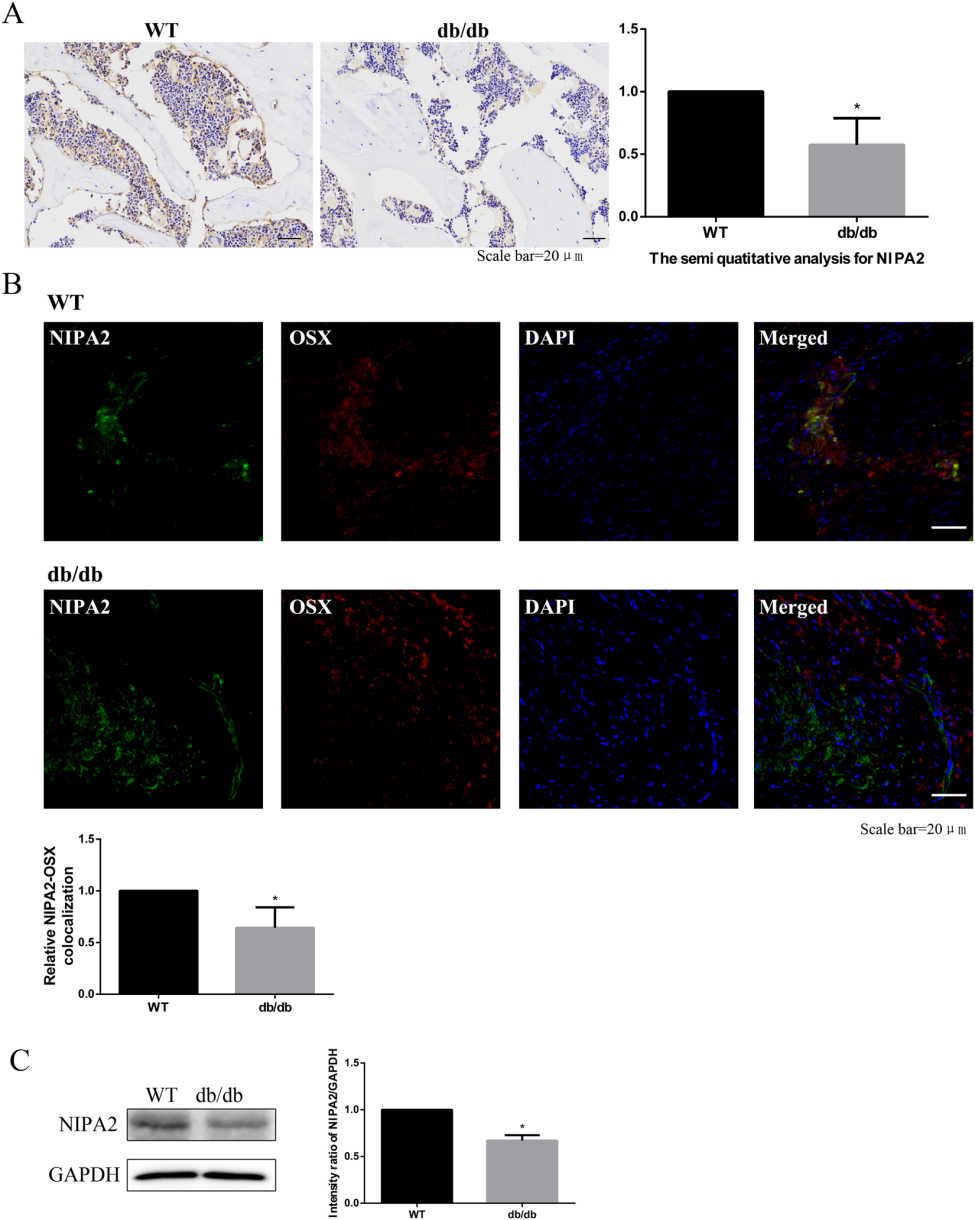
*In vivo* downregulation of NIPA2 in the bone tissues and osteoblasts of type 2 diabetes osteoporosis mice. C57BKS db/db mice were included in the db/db group (n = 15), and their lean littermates C57BKS mice were included in the WT group (n = 15). **(A)** IHC staining showing that the NIPA2 expression at 12 weeks was significantly lower in the db/db group than in the WT group. **(B)** IF showing that the colocalization of NIPA2 and Osx was significantly lower in the db/db group than in the WT group. **(C)** Western-blot of bone tissues showing the expression of NIPA2 in db/db group was significantly lower than in the WT group. Data are expressed as the fold induction relative to the control. Values are presented as the mean ± SD. *P < 0.05 vs. WT, n = 15 per group.

IHC analysis was used to detect the level of NIPA2 protein expression in mouse bone tissue, revealing significantly lower NIPA2 protein levels in the db/db group than in the WT group (Fig. 2A). We next examined the colocalization of NIPA2 and a biomarker of osteoblasts, Osx^23,24^. The results showed that the colocalization of NIPA2 and Osx was lower in the db/db group than in the WT group (Fig. 2B). Western blot of bone tissue showed that the expression of NIPA2 protein was lower in the db/db group than in the WT group (Fig. 2C). These results suggested that the expression of NIPA2 in osteoblasts was downregulated in type 2 diabetes osteoporosis models.

### NIPA2 and mitophagy in primary culture osteoblasts

**Figure 3:**
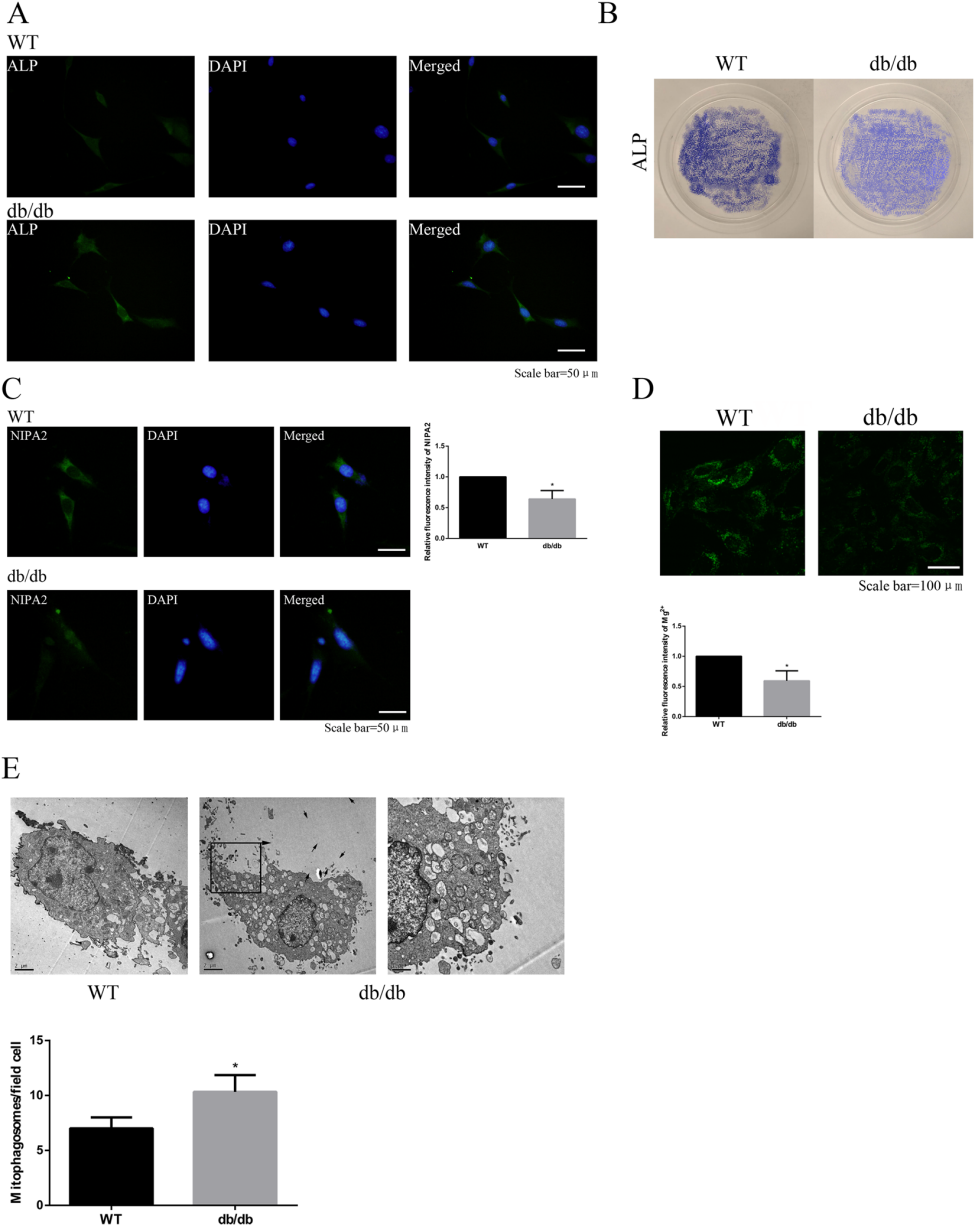
NIPA2 expression and mitophagy in primary osteoblasts. **(A)** Immunofluorescence showing ALP expression in primary osteoblasts of each group. **(B)** ALP staining images of primary osteoblasts in WT and db/db groups. **(C)** Immunofluorescence of NIPA2 in primary osteoblasts of WT and db/db groups (values are presented as the mean ± SD; *P < 0.05 vs. WT, n = 30 cells from three independent experiments). **(D)** Detection of magnesium fluorescence in primary osteoblasts of WT and db/db groups (values are presented as the mean ± SD; *P < 0.05 vs. WT, n = 30 cells from three independent experiments). **(E)** TEM shows the number of mitophagosomes in primary osteoblasts of WT and db/db groups (scale bars, 1 μm and 2 μm. n = 3 per group. Values are presented as the mean ± SD *P < 0.05 vs. WT).

Immunofluorescence and staining of ALP showed successful of primary osteoblast isolation of the WT and db/db groups (Fig. 3A,B). Lower fluorescence intensity of NIPA2 was found in the db/db groups than in the WT groups (Fig. 3C). At the same time, lower intracellular magnesium was found in the db/db groups too (Fig. 3D). The number of mitophagosomes was significantly increased in the db/db groups than in the WT groups, which showed a higher level of mitophagy in the db/db groups (Fig. 3E).

### Effects of high glucose on NIPA2 expression, intracellular Mg^2+^ and mitophagy ***in vitro***

**Figure 4:**
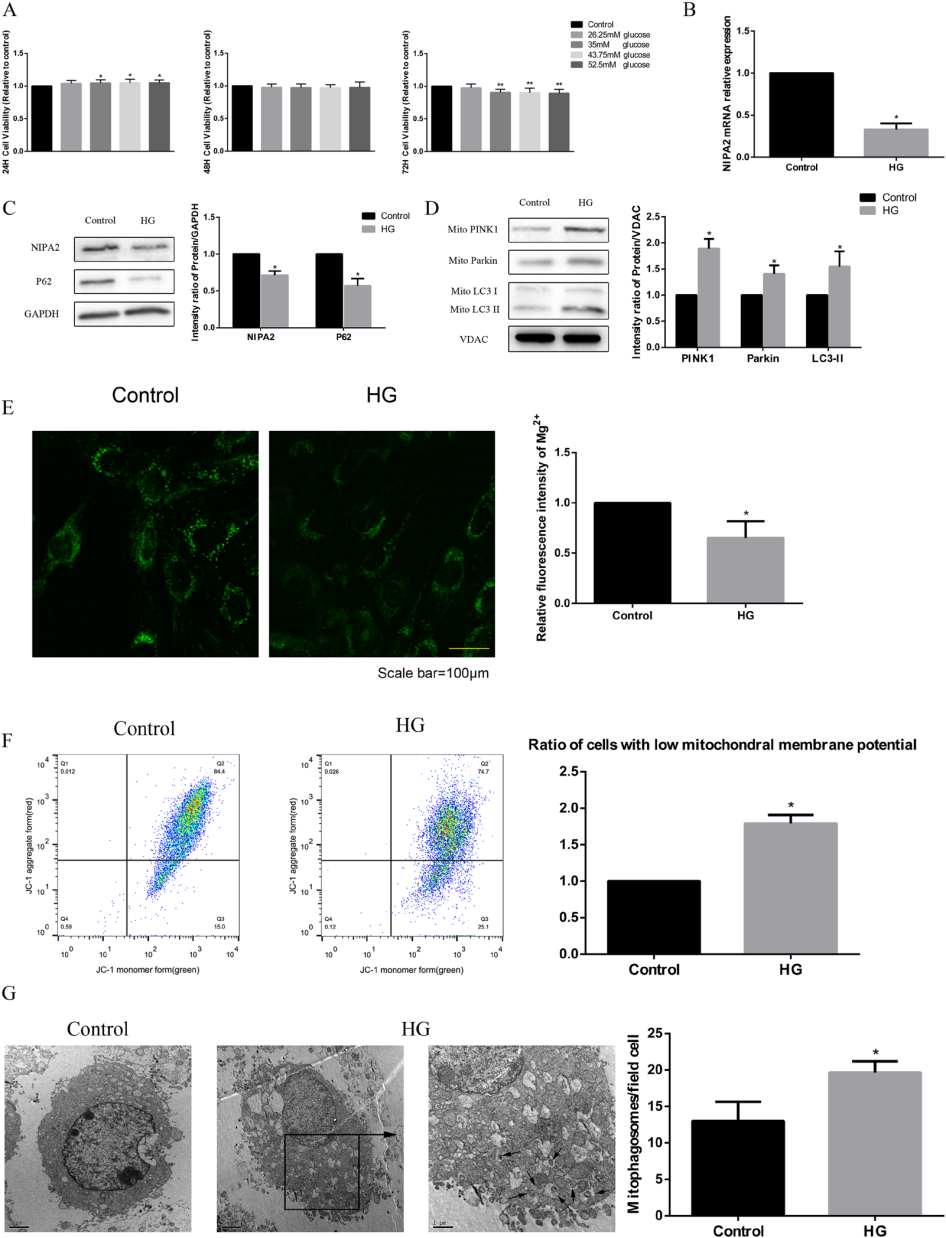
Effects of high glucose on NIPA2 expression, intracellular Mg^2+^ and mitophagy *in vitro*. **(A)** Cells were treated with various concentrations of glucose for 24 h, 48 h and 72 h. Cell viability was estimated using the CCK-8 assay. Values represent the mean ± SD of three independent experiments. *P < 0.05 vs. control. Data are expressed as the fold induction relative to the control. **(B)** RT-PCR analysis of NIPA2 mRNA expression in hFOB1.19 cells treated with normal medium (control, 17.5 mM) and high glucose (HG, 35 mM) for 72 h. Values are presented as the mean ± SD of three independent experiments. *P < 0.05 vs. control. **(C)** Western blot showing NIPA2 and P62 protein levels in hFOB1.19 cells of the control and HG groups at 72 h. Values represent the mean ± SD of three independent experiments. *P < 0.05 vs. control. **(D)** Western blot showing PINK1, Parkin and LC3 protein levels in the mitochondria of hFOB1.19 cells of the control and HG groups at 72 h. Values represent the mean ± SD of three independent experiments. *P < 0.05 vs. control. **(E)** Detection of Mg^2+^ fluorescence after Mag-fura-2 AM staining at 72 h. The intracellular Mg^2+^ was significantly decreased in the HG group compared with that in the control group (values are presented as the mean ± SD; *P < 0.05 vs. control, n = 30 cells from three independent experiments; scale bar, 100 μm). **(F)** Detection of MMP using flow cytometry at 72 h, revealing a significantly higher number of cells with low MMP in the HG group than in the control group (values are presented as the mean ± SD of three independent experiments. *P < 0.05 vs control). The above data are expressed as the fold induction relative to the control. **(G)** TEM results at 72 h. The number of mitophagosomes was significantly higher in the HG group than in the control group (scale bars, 1 μm and 2 μm. n = 3 per group. Values are presented as the mean ± SD *P < 0.05 vs. control).

We determined the optimal concentration and time (35 mM glucose and 72 h treatment) of HG treatment in this study in a pre-experiment using the CCK-8 cell viability assay (Fig. 4A). Then we found that HG reduced both the protein and gene expression of NIPA2 in hFOB1.19 cells (Fig. 4B,C). At the same time, lower intracellular Mg^2+^ was found in the HG groups than in the control (Fig. 4E). The MMPs of osteoblasts in the HG groups were significantly decreased (Fig. 4F). Western blot analysis of mitophagy-associated proteins showed upregulated LC3-II, PINK1 and Parkin but downregulated P62 in the HG group (Fig. 4C,D). The effect of glucose on mitophagy was confirmed by TEM, which was used to count the number of mitophagosomes and is considered the gold standard for monitoring mitophagy. The number of mitophagosomes was significantly increased in the HG group compared to that in the control group (Fig. 4G). These results suggest that an HG concentration reduces the protein and mRNA expression of NIPA2. HG concentrations also reduced intracellular Mg^2+^ and induced mitophagy.

### Effects of NIPA2 on mitophagy

**Figure 5:**
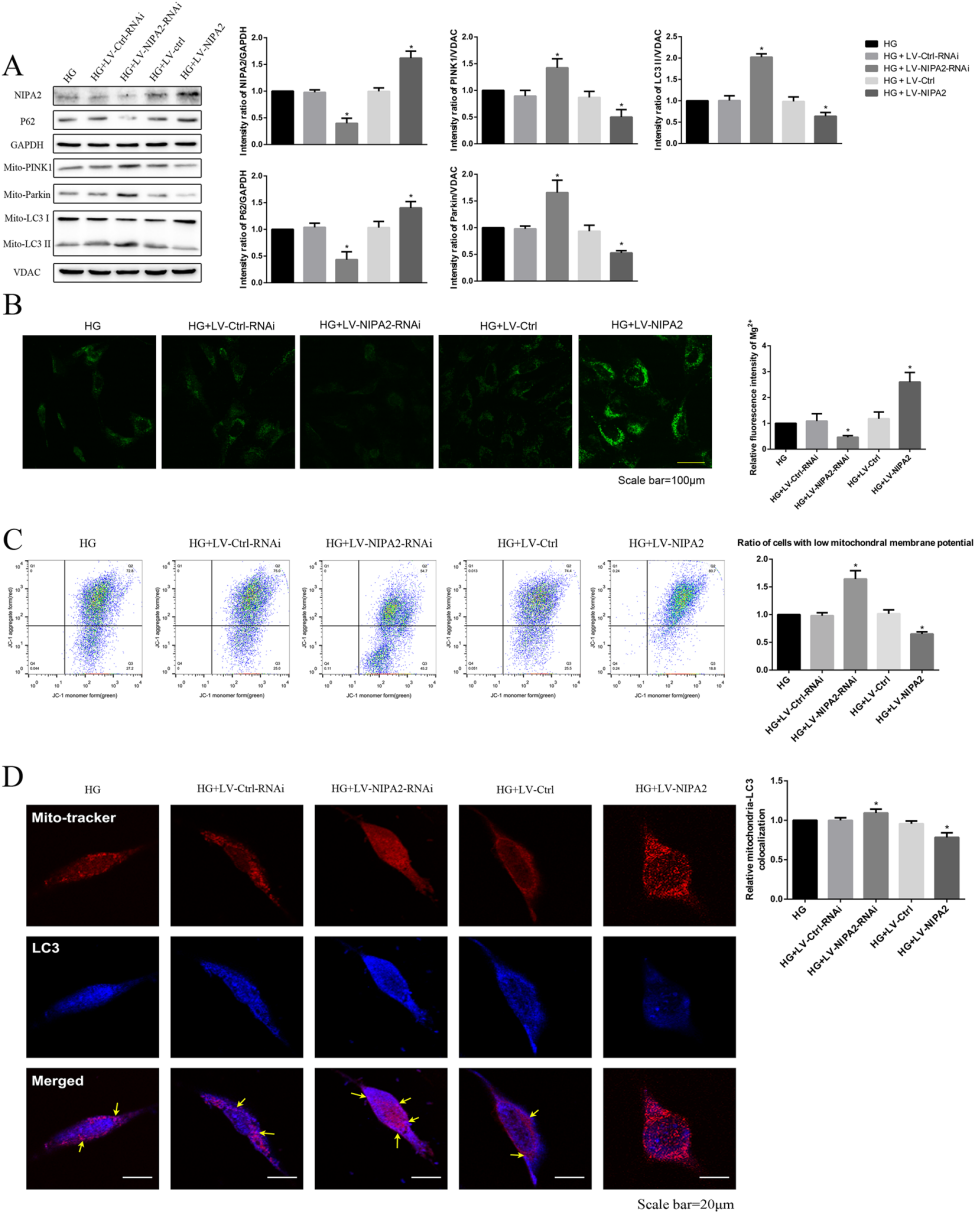
Effects of NIPA2 on mitophagy. **(A)** Western blot showing the NIPA2, P62, mitochondrial PINK1, Parkin and LC3 protein levels in hFOB1.19 cells after transfection and HG treatment for 72 h. **(B)** Detection of Mg^2+^ fluorescence after successful NIPA2 transfection in HG at 72 h. **(C)** Flow cytometry showing the MMP of transfected hFOB1.19 cells after HG treatment for 72 h. Values are presented as the mean ± SD of three independent experiments. *P < 0.05 vs. HG. **(D)** Quantitative analysis of transfected hFOB1.19 cells treated with HG for 72 h that demonstrate colocalization of LC3 dots with mitochondria per cell (*P < 0.05, n = 9 cells from three independent experiments; Scale bar, 20 μm, values are presented as the mean ± SD). Data are expressed as the fold induction relative to HG.

First, we successfully overexpressed (HG + LV-NIPA2) and silenced (HG + LV-NIPA2-RNAi) NIPA2 *in vitro* (Fig. 5A). In our study, overexpression of NIPA2 under HG treatment increased the concentration of intracellular Mg^2+^, and downregulation of NIPA2 yielded the opposite result (Fig. 5B).

Upon comparing the HG groups, the MMP of NIPA2-overexpressing cells was increased in the HG environment, while silencing NIPA2 had the opposite effect (Fig. 5C). Furthermore, in the presence of HG, the mitochondrial levels of LC3-II, PINK1 and Parkin were upregulated in NIPA2-silenced cells and downregulated in NIPA2-overexpressing cells compared to those in the HG group (Fig. 5A). To provide more solid evidence for the role of NIPA2 in mitophagy, confocal laser scanning microscopy was used to examine the lentivirus-transfected hFOB1.19 cells. The results shown in Fig. 5D illustrate that the accumulation of LC3 in mitochondria was increased in the HG plus NIPA2-silenced group compared to that in the HG group, while the opposite result was shown in the HG plus NIPA2-overexpressing group. Taken together, these results strongly suggest that in the presence of HG, NIPA2 might negatively correlate with mitophagy by regulating intracellular Mg^2+^.

### Effects of NIPA2 on osteogenic capability by regulating osteoblast mitophagy

**Figure 6:**
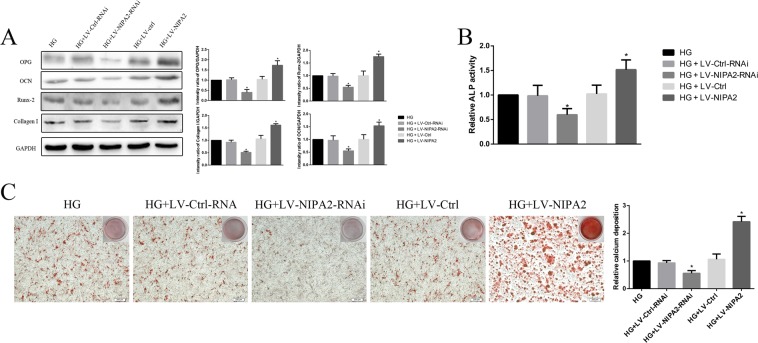
Effects of NIPA2 on osteogenic capability. **(A)** Western blot showing OPG, OCN, Runx-2 and Collagen-I protein levels in transfected hFOB1.19 cells after HG treatment for 72 h. **(B)** Activity of ALP in transfected hFOB1.19 cells after HG treatment for 72 h. **(C)** Alizarin red S staining assay showing the mineralized extracellular matrix of transfected hFOB1.19 cells after osteogenic differentiation for 14 days. Values are presented as the mean ± SD of three independent experiments. *P < 0.05 vs. HG, ^#^P < 0.05 vs. HG + LV-NIPA2. Data are expressed as the fold induction relative to HG.

We next assessed the osteoblast osteogenic capability-associated proteins OPG, OCN, Runx-2, Collagen-I and the activity of ALP. The levels of both OPG, OCN, Runx-2 and Collagen-I were upregulated in the HG plus NIPA2-overexpressing group compared to those in the HG group, while the opposite trend was observed in the HG plus NIPA2-silencing group (Fig. 6A). The results of ALP activity also showed the same tendency (Fig. 6B). The alizarin red S staining assay was also used to measure the effect of NIPA2 on the ability of hFOB1.19 cells to differentiate into mature osteoblasts and form a mineralized extracellular matrix. After 14 days of culture, the group treated with a combination of HG and NIPA2 overexpression exhibited a higher level of mineralized nodule formation than the HG groups, and the opposite result was observed in the HG plus NIPA2-silencing groups (Fig. 6C). These results suggested that in the presence of HG, NIPA2 might be positively correlated with osteogenic capability.

**Figure 7:**
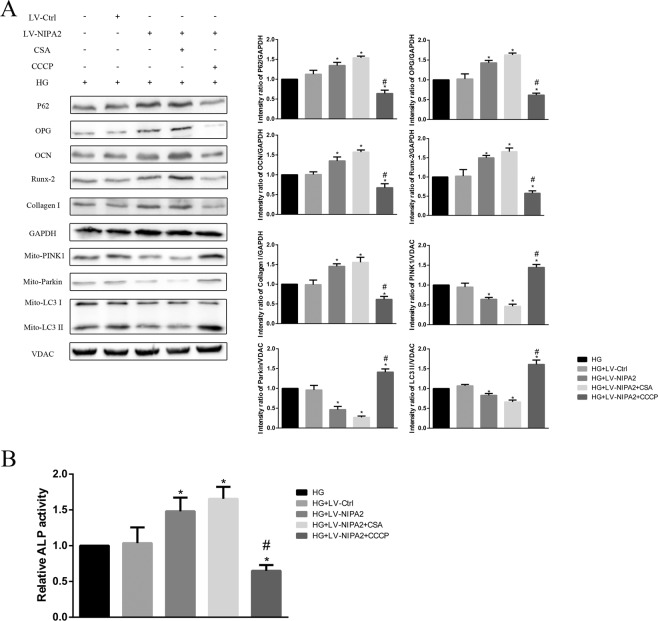
Effects of NIPA2 mediated mitophagy on osteogenic capability. **(A)** Western blot showing P62, OPG, OCN, Runx-2 Collagen-1 and mitochondrial PINK1, Parkin, LC3 protein levels in NIPA2-overexpressing hFOB1.19 cells after 0.5 μM CsA or 10 μM CCCP treatment in HG at 72 h. **(B)** Activity of ALP in NIPA2-overexpressing hFOB1.19 cells after 0.5 μM CsA or 10 μM CCCP treatment in HG at 72 h. Values are presented as the mean ± SD of three independent experiments. *P < 0.05 vs. HG, ^#^P < 0.05 vs. HG + LV-NIPA2. Data are expressed as the fold induction relative to HG.

We then evaluated the relationship between mitophagy and osteogenic capability by up and down regulating mitophagy. CsA and CCCP are known to function as inhibitors and activators of mitophagy, respectively, by regulating the membrane potential of mitochondria^25,26^. After treatment for 72 h, we examined the mitophagy-associated proteins LC3-II, PINK1, Parkin and P62 and the osteogenic capability biomarkers OPG, OCN, Runx-2 and Collagen-I, and also the activity of ALP. Both the NIPA2-overexpressing group (HG + LV-NIPA2) and the NIPA2-overexpressing plus CsA group (0.5 μM, HG + LV-NIPA2 + CsA) had lower LC3-II, PINK1 and Parkin expression levels than the HG groups, while the P62 expression trend was opposite. The reduction of mitophagy was effectively rescued by CCCP treatment (10 μM, HG + LV-NIPA2 + CCCP) (Fig. 7A). In contrast, the expression of OPG, OCN,Runx-2, Collagen-I and ALP activity were increased in the HG + LV-NIPA2 and HG + LV-NIPA2 + CsA groups, while opposite results were observed in the HG + LV-NIPA2 + CCCP groups (Fig. 7A,B).

**Figure 8:**
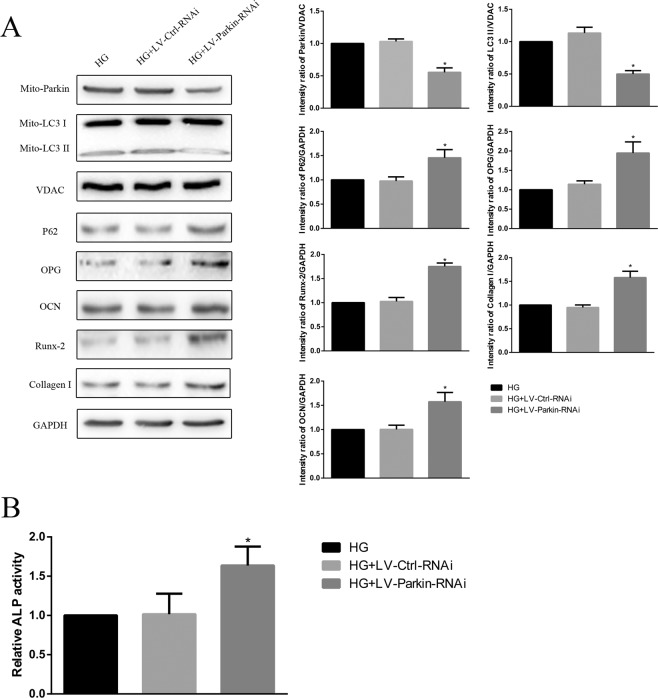
**(A)** Western blot showing P62, OPG, OCN, Runx-2 Collagen-I and mitochondrial Parkin, LC3 protein levels of Parkin-knockdown hFOB1.19 cells after HG treatment for 72 h. **(B)** Activity of ALP in Parkin-knockdown hFOB1.19 cells after HG treatment for 72 h. Values are presented as the mean ± SD of three independent experiments. *P < 0.05 vs. HG, ^#^P < 0.05 vs. HG + LV-NIPA2. Data are expressed as the fold induction relative to HG.

After we successfully knocked down the expression of Parkin (HG + LV-Parkin-RNAi, Fig. 8A), downregulation of LC3-II and upregulation of P62 were observed in comparison with those in the HG alone groups. The expression levels of the osteogenic capability biomarkers OPG, OCN, Runx-2, Collagen-I and ALP activity were downregulated in the HG + LV-Parkin-RNAi groups (Fig. 8A,B).

These data suggested that in an HG environment, a negative correlation may exist between osteoblast mitophagy and osteogenic capability.

### The mitophagy of hFOB1.19 cells regulated by NIPA2 was mediated via the PGC-1α/FoxO3a signaling pathway

**Figure 9:**
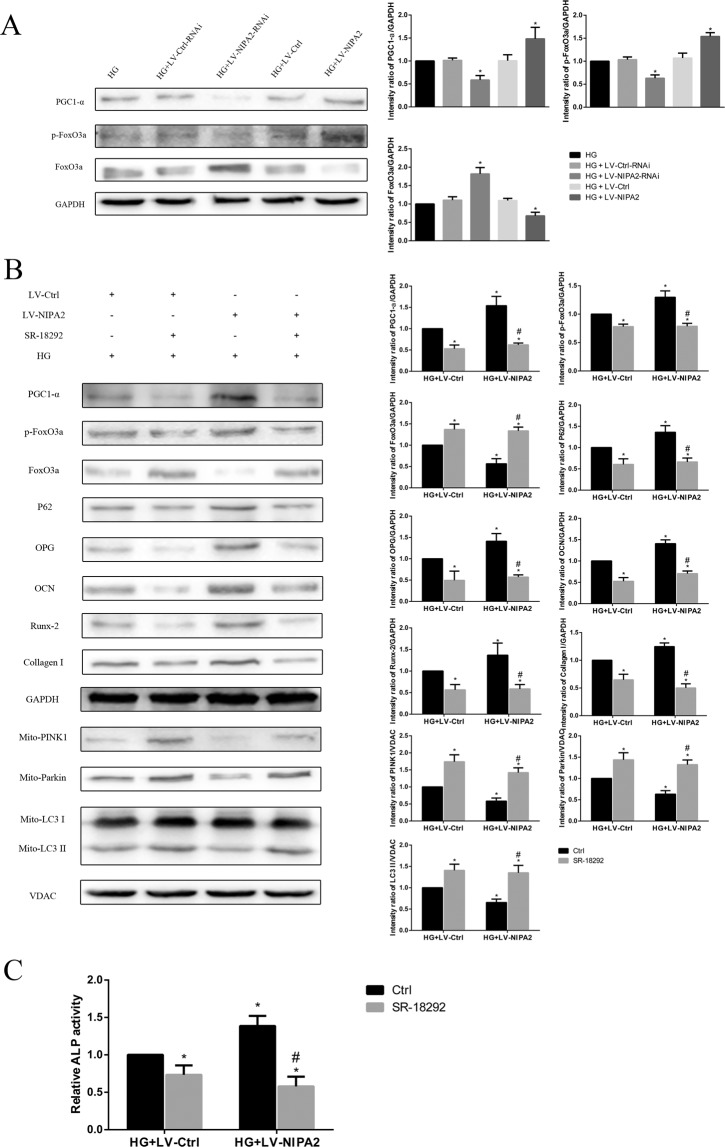
The mitophagy of hFOB1.19 cells regulated by NIPA2 was mediated via the PGC-1α/FoxO3a signaling pathway. **(A)** Western blot showing PGC-1α, p-FoxO3a and FoxO3a protein levels in NIPA2-transfected hFOB1.19 cells after HG treatment for 72 h. *P < 0.05 vs. HG. Data are expressed as the fold induction relative to HG. **(B)** Western blot showing PGC-1α, p-FoxO3a, FoxO3a, P62, OPG, OCN, Runx-2, Collagen-I and mitochondrial PINK1, Parkin, and LC3 protein levels in NIPA2-overexpressing hFOB1.19 cells after 10 μM SR-18292 treatment in HG for 72 h. **(C)** Activity of ALP in NIPA2-overexpressing hFOB1.19 cells after 10 μM SR-18292 treatment in HG for 72 h. *P < 0.05 vs. HG + LV-Ctrl, ^#^P < 0.05 vs. HG + LV-NIPA2. Data are expressed as the fold induction relative to HG + LV-Ctrl. Values are presented as the mean ± SD of three independent experiments.

We then sought to clarify the mechanism by which NIPA2 regulates mitophagy. In our study, significant PGC-1α and p-FoxO3a downregulation was observed in the HG + LV-NIPA2-RNAi groups, and this downregulation was rescued by overexpression of NIPA2 (Fig. 9A). The FoxO3a expression trend was opposite those of PGC-1α and p-FoxO3a (Fig. 9A). SR-18292 (10 μM), a PGC-1α inhibitor, inhibited the effects of NIPA2 overexpression on the PGC-1α/FoxO3a pathway (PGC-1α, FoxO3a and p-FoxO3a), mitophagy-associated proteins (P62, LC3-II, PINK1 and Parkin), osteogenic capability biomarkers (OPG, OCN, Runx-2 and Collagen-I) and ALP activity (Fig. 9B,C). These results indicated that NIPA2 might regulate mitophagy via the PGC-1α/FoxO3a signaling pathway.

### Effects of NIPA2 and mitophagy ***in vivo***

**Figure 10:**
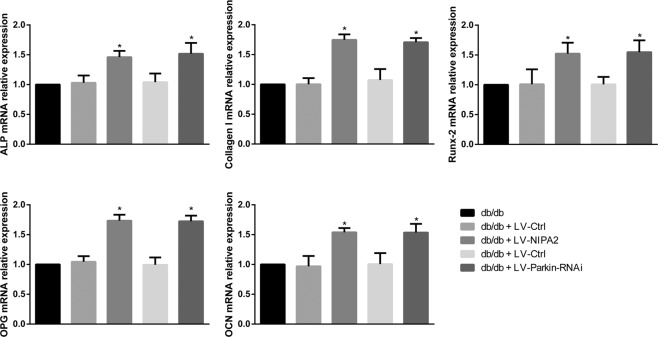
RT-PCR analysis of ALP, Collagen-I, Runx-2, OPG, and OCN mRNA levels in the bone tissues of db/db mice after overexpression of NIPA2 or knockdown of Parkin. *P P < 0.05 vs. db/db. Data are expressed as the fold induction relative to db/db.

We then examined the mRNA levels of osteogenic capability biomarkers OPG, OCN, Runx-2 Collagen-I and ALP in the bone tissue after overexpression of NIPA2 or knockdown of Parkin in db/db mice. We found that overexpression of NIPA2 and knockdown of Parkin in db/db mice might up-regulate the osteoblast function (Fig. 10). These results showed the positive effects of NIPA2 and the negative effects of mitophagy *in vivo*.

## Discussion

We found significant NIPA2 downregulation in db/db mice and in primary culture osteoblast-derived from db/db mice. *In vitro*, both the mRNA and protein levels of NIPA2 were significantly reduced by HG concentration treatment. In addition, NIPA2 could positively regulate the osteogenic capacity of hFOB1.19 osteoblasts by inactivating PINK1/Parkin-mediated mitophagy via the PGC-1α/FoxO3a/MMP pathway.

As a widely used T_2_DM model, the specific mechanism of the reduced bone formation in db/db mice remains conflicting. Downregulation of the highly selective magnesium transporter NIPA2 in bone tissues and osteoblasts of type 2 diabetes osteoporosis was highlighted for the first time in our *in vivo* experiments. The phenomenon was also confirmed by primary cultured osteoblasts in our study. To the best of our knowledge, this is the first study to demonstrate an association between NIPA2 and type 2 diabetes osteoporosis using animal models.

The effects of HG on divalent metal ion transporters have been widely reported. Zhang *et al*. reported that zinc transporter 7 (ZnT7) was elevated by HG treatment in rat peritoneal mesothelial cells^27^. Khan *et al*. found upregulation of the iron transporters DMT1 and IREG1 in response to high levels of glucose in human umbilical vein endothelial cells^28^. Although magnesium is mostly stored in bones and has been identified as a risk factor of osteoporosis, to our knowledge, no study has confirmed the role of magnesium transporters in type 2 diabetes osteoporosis. Our study provides the first evidence that HG downregulates the mRNA and protein levels of the magnesium transporter NIPA2 in osteoblasts.

The role of autophagy in the pathological process of osteoporosis is controversial. Lin *et al*. found that both deletion of the autophagy-related gene ATG7 and pharmacological inactivation of autophagy ameliorated bone loss in osteoporosis rats^29^. In contrast, Li *et al*. suggested that defective autophagy in osteoblasts induces endoplasmic reticulum stress and causes osteoporosis^30^. However, the intracellular location of autophagy was not investigated in these studies. Unlike in previous studies, we found that osteogenic dysfunction in hFOB1.19 cells was accelerated by mitophagy under HG treatment and the function of osteoblasts was rescued by both pharmacological and genetic inhibition of mitophagy. Our study provides a new direction to further investigate the effect of autophagy on type 2 diabetes osteoporosis, as we showed that autophagy occurring in different organelles may have specific biological effects. Furthermore, we also confirmed that the level of mitophagy in the HG environment was negatively regulated by NIPA2. The positive effects of NIPA2 and the negative effects of mitophagy were also confirmed through our *in vivo* experiments.

The downregulating effect of high magnesium on mitophagy has been previously reported^31^, but the specific mechanism is still unclear. Magnesium ion stimulation may enhance the osteogenic activity of bone marrow stromal cells by upregulating the expression of PGC-1α according to a previous study^32^. Kang *et al*. suggested that overexpression of PGC-1α attenuates mitophagy via deactivating (phosphorylating) FoxO3a in muscle disuse atrophy^33^. The activation of FoxO3a may decrease MMP by activating BIM and increasing the permeability of the mitochondrial membrane^34^. In our study, overexpression of NIPA2 under HG treatment resulted in the amelioration of PGC-1α reduction and inactivation of FoxO3a. PINK1 accumulates on the outer membrane of mitochondria devoid of membrane potential, and Parkin activation is mediated by the phosphorylation of Ser65 in the ubiquitin PINK1 and in the Parkin Ubl domain^35,36^. Once Parkin localizes to mitochondria, P62 and LC3 accumulate at the mitochondrial membrane, driving the recruitment of autophagosomal membranes^37^. In our study, NIPA2 increased the MMP and attenuated the PINK1/Parkin-mediated mitophagy of hFOB1.19 cells by regulating intracellular Mg^2+^. Considering these results together, we suggest that NIPA2 regulates PINK1/Parkin-mediated osteoblast mitophagy via the PGC-1α/FoxO3a/MMP pathway.

Although the db/db mouse is a widely used type 2 diabetes model. The mechanism of osteoporosis in db/db mice is complicated, such as hyperglycemia, AGEs, abnormality of renin-angiotensin system, and leptin. AGEs mediated oxidative stress and apoptosis in osteoblasts lead to loss of bone mass in db/db mice^38^. Abnormal expression of components of renin-angiotensin system and bradykinin receptor-2 in bone tissue also lead to osteoporosis in db/db mice. Leptin controls bone formation through a central pathway following binding to its specific receptors located on hypothalamic nuclei^39^. But the specific mechanism of high glucose and osteoporosis is still unknown. Therefore, we found absent NIPA2 and excessive mitophagy in db/db mice and then examined the effect of NIPA2 and mitophagy on hFOB1.19 cells in a high glucose environment. The observations of mitophagy and osteogenic capabilities at different intracellular Mg^2+^ concentrations were included in our experiments, we did not investigate the specific mechanism immediately regulated by Mg^2+^. However, the regulation of PGC-1α by Mg^2+^ has been confirmed by many researchers. Yoshizawa *et al*. found enhanced PGC-1α expression in the presence of Mg^2+^ in bone marrow stromal cells^40^. An *in vivo* experiment also confirmed the positive effect of Mg^2+^ on the expression of PGC-1α^41^. Consequently, our existing data demonstrate the specific molecular mechanism regulated by intracellular Mg^2+^.

## Materials and Methods

### Experimental animals

The male diabetic db/db mice and wild type mice(lean littermates C57BKS mice) were bred under temperature conditioner at 22.6 ± 2 °C with a 12 hours day/night cycle and aged 8 weeks. Fifteen mice were placed into each group. The diabetic db/db mice are as the db/db group and the wild type mice are as the control. Mice tibias were collected and cut into pieces, then ground into powder in liquid nitrogen. These mice were purchased from Biomedical Research Institute of Nanjing University (Jiangsu, China). This study was approved by the Institutional Review Board of the First Hospital of China Medical University and conformed to the National Institutes of Health Guide for the Care and Use of Laboratory Animals.

### Plasma measurements

Blood samples were collected from the animals’ tail veins. The samples were used for measuring the fasting blood glucose (FBG) levels (Roche blood glucose instrument, Roche, Basel, Switzerland). Radioimmunoassay (3v-diagnostic Bioengineer, Shandong, China) was used for the fasting plasma insulin (FINS) analysis, while ELISA (Rat Estrogen/E ELISA Kit, 3v-diagnostic Bioengineer) was used for the plasma estrogen analysis. The insulin sensitivity index (ISI) was calculated as the -ln(FINS•FPG)^42^.

### Micro-CT scan

For measurement of the bone microstructures of the animals, mice were killed when their skeleton maturate at 12 weeks of age according to previous study^40^. Their tibias were aseptically removed and assessed by Micro-CT scan. The following scan parameters were set as :1024 × 1024 image matrix, 80 kV voltage, 80 μA current, 2.96 s exposure time. The actual section thickness of a cancellous bone area from the distal growth plate was 1.0 mm by 3.0 mm. The 190 image extractions was used to make a line of reconstruction. Images were made via micro-CT, and the following parameters were defined as: bone mineral density (BMD), trabecular bone volume per tissue volume (BV/TV), trabecular number (Tb.N), and trabecular thickness (Tb.Th).

### Haematoxylin and eosin (HE) staining

For HE staining analysis, mice tibia tissues were fixed in 4% paraformaldehyde. After fixation, the samples were decalcified in 10% EDTA for 21 days before embedded in paraffin and then collected with a systematic series of 16 μm sections by microtome. Standard HE staining was performed and photographed. The average number of deposits per section was calculated using Image-Pro Plus 6.0 (Media Cybernetics, USA).

### Immunohistochemistry

After fixation, decalcification embedded and sectioned as mentioned above, Mice tibia sections (10-μm thick) were deparaffinized in xylene. Rehydrated sections were obtained using a graded series of ethanol, and then the sections expose antigen epitope. The peroxidase activity was quenched for 15 min in 3% H_2_O_2_. After washing, the sections were incubated for 30 min in goat serum at 37 °C. They were then incubated overnight in primary rabbit monoclonal anti-NIPA2 antibody (1:100; catalog number: NBP2-49510; Novus, USA) at 4 °C. And next, secondary goat anti-rabbit antibody was applied for 45 min at 37 °C. Sections were processed with an ABC working solution (Zsbio) for 25 min at 37 °C and developed with 3,3-diaminobenzidine (DAB; Zsbio). When microscopically observing brown particles, the slices were washed in distilled water, and counterstained using hematoxylin. Every ten fields of each sample were included. Immunohistochemical quantitative analysis of NIPA2 expression was performed by using Image-Pro Plus 6.0. And each visual field was measured with a mean density value which was described as integrated optical density (IOD) divided by the relevant area.

### Isolation and culture of primary osteoblasts

Mice tibias without muscle, soft tissue and periosteum were prepared and then removed bone marrow cells by flushing with α-MEM. Osteoblasts on the surface of bone were then collected^43^. Primary osteoblasts were cultured in α-MEM containing 10% FBS for 7 days before experiments. Immunofluorescence and stained of ALP were used to evaluated the isolation of primary osteoblasts^44^.

### Cell culture and materials

The hFOB1.19 cells were routinely cultured at 33.5 °C and in a 1:1 mixture medium of Ham’s F12 and Dulbecco’s Modified Eagle’s (DMEM/F12) supplemented with 17.5 mM glucose (HyClone, Utah, USA), 10% fetal bovine serum (HyClone) and 0.3 g/L G418 (Sigma-Aldrich, Darmstadt, Germany) in a humidified 5% CO_2_ atmosphere. Trypsin-EDTA (HyClone) was used for cell passage. Before experiments, the hFOB1.19 cells were plated at 10^4^ cells/cm^2^. Cells were cultured in osteogenic medium to which was added: DMEM/F12,10 mM β-glycerol phosphate (Sigma-Aldrich), 50 μg/ml ascorbic acid (Sigma-Aldrich) in order to test osteogenic differentiation. Cells were maintained in 5% CO_2_ at 33.5 °C for 14 days and treated with 35 mM glucose before HG use.

Carbonyl cyanide 3-chlorophenylhydrazone (CCCP) and SR-18292 were both provided from MCE (New Jersey, USA), and cyclosporine A (CsA) was obtained from Solarbio (Beijing, China). Bovine serum albumin (BSA) was obtained from Sigma-Aldrich. Anti-microtubule-associated protein 1 A/1B-light chain 3 (LC3, ab48394), anti-osteoprotegerin (OPG, ab73400), anti-osteocalcin (OCN, ab13420), anti-Runx-2 (ab76956), anti-Collagen I (ab34710), Alexa 488(ab150077) and Alexa 647(ab150115) were purchased from Abcam (Cambridge, UK). Anti-PINK1 (23274-1-AP), anti-Parkin (14060-1-AP) and anti-mitochondrial outer membrane protein porin of 36 kDa-like (VDAC, 10866-1-AP) were purchased from Proteintech (Hubei, China). Anti-sequestosome 1 (SQSTM1/P62, #39749), anti-PGC-1α (#2178), anti-FoxO3a (#12829), and anti-phospho-FoxO3a (p-FoxO3a, #9466) were obtained from Cell Signaling Technology (Massachusetts, USA). Anti-glyceraldehyde-3-phosphate dehydrogenase (GAPDH, TA309157) and all the secondary antibodies were provided by Zsbio.

### Cell viability analysis

For the CCK-8 assay measurement of cell viability, the cells were performed as described below. Before treatment, the cells were plated on 96-well plates for 24 h. After a series of HG treatment (24 h, 48 h, 72 h),each well was added with CCK-8 solution and incubated for 1 h. Absorbance was determined with a microplate reader(Thermo, Massachusetts, USA) at 450 nm. The results are performed with percentages while the untreated cells was taken into 100%.

### Transmission electron microscopy (TEM)

T The cells were scraped after treatment, collected by centrifugation, washed with PBS and fixed with 5% glutaraldehyde. After the cells were dehydrated, embedded, sectioned, stained, the mitophagosomes were observed by TEM. The quantity of mitophagosomes was calculated in 10 fields a view.

### Real-time reverse transcription (RT)-PCR

For detection of real-time PCR, total RNA was isolated by MiniBEST universal RNA extraction kit (Takara, Shiga, Japan). And reverse transcribtion was performed by Primescript RT master mix (Takara). LightCycler 480 real-time PCR system (Roche) with a SYBR premix ex taq II kit (Takara) is used to test real-time PCR. NIPA2, ALP, Runx-2, Collagen-I, OPG, OCN and β-actin listed in Table 1 were subjected to amplify the primers under the universal amplificated conditions. Quantification of mRNA expression was performed compared with the cycle threshold (Ct) values while the β-actin was used as the internal control. The experimental data were processed by the 2^−ΔΔCt^ method as follows: ΔΔCt = (Ct target − Ct internal control) experiment group − (Ct target − Ct internal control) control group.

**Table 1:** Primer sequences used in real- time PCR experiments.

Gene	Primer sequence 5′-3′
NIPA2	F: CAGTTATGGTCATTCATGCTCCAA
	R: TTAATATCAAGGCCACAATGACCAC
Collagen-I	F: AGAGCTTCGGCAGCAGGA
	R: CTTATAGCAGTTCTGCCTGC
ALP	F: AACATCAGGGACATTGACGTG
	R: GTATCTCGGTTTGAAGCTCTTCC
OPG	F: GCGCTCGTGTTTCTGGACA
	R: AGTATAGACACTCGTCACTGGTG
OCN	F: CACTCCTCGCCCTATTGGC
	R: CCCTCCTGCTTGGACACAAAG
Runx-2	F: CCT TCCAGACCAGCAGCAG
	R: TCCGTCAGCGTCAACACCA
β-actin	F: GACAGGATGCAGAAGGAGATTACT
	R: TGATCCACATCTGCTG GAAGGT

For RNA extraction from bone tissue, tibia of mice was dissected out, and removed non-osseous tissue with ice-cold PBS, then flash-frozen in liquid nitrogen. Bone was then minced in Trizol, and then homogenized. After centrifugation at 15000 g for 10 min, the supernatant was used for RNA isolation using the RNeasy MinElute Cleanup kit (Qiagen).

### Western blot analysis

After treatment, the cells were washed with ice-cold PBS and prepared in lysis buffer (50 mM Tris-HCl, 150 mM NaCl, 1% NP-40, 0.5% sodium deoxycholate, 0.1% sodium dodecyl sulfate) containing protease and phosphorylase inhibitor cocktails. After centrifugation at 12000 g for 30 min at 4 °C, the supernatant containing total protein was collected. Mitochondria-associated proteins were separated by a cell mitochondria isolation kit (Beyotime, Shanghai, China) according to the manufacturer’s instructions, and the protein concentration was determined using a BCA protein concentration assay kit (Boster, Hubei, China). The samples were separated by SDS-PAGE and then transferred to polyvinylidene difluoride (PVDF) membranes (Millipore, Massachusetts, USA) (210 mA, between 30 and 120 min according to the molecular mass of the protein). After blocking for 2 h with 5% nonfat milk, the PVDF membranes were incubated with the appropriate primary antibodies (1:500 or 1:1000 dilution) at 4 °C overnight. Then, the blots were incubated with anti-mouse or anti-rabbit IgG conjugated to horseradish peroxidase (1:5000 dilution) for 1 h at room temperature. Immunoreactive bands were visualized by the EC3 imaging system (UVP Inc., California, USA), and the optical density of each band was measured with ImageJ software (NIH, USA). In these experiments, GAPDH and VDAC were used as loading controls for the whole cellular and mitochondrial proteins, respectively. The rates between the proteins of interest and loading controls of the same sample were calculated as relative content and expressed graphically. The results were averaged from three independent experiments.

### Measurement of mitochondrial membrane potential (MMP, Δψm)

The cellular MMP was measured using a JC-1 mitochondrial membrane potential assay kit (C2006, Beyotime). JC-1 shows red fluorescent aggregates in the mitochondrial matrix, and when the membrane potential is lost, these aggregates break up into green fluorescent monomers. Briefly, cells were obtained and washed with ice-cold PBS and then incubated with JC-1 for 45 min at 37 °C in the absence of light. The cells were then analyzed by bivariate flow cytometry on a FACS SCAN flow cytometer (BD Biosciences, California, USA). Mitochondrial depolarization was indicated by the percentage of green fluorescence intensity. Data were averaged from three independent experiments.

### Measurement of intracellular Mg^2+^

For detection of intracellular Mg^2+^, cells were incubated with Mag-fura-2 AM (5 μM) for 30 min, followed by meansuring the fluorescence of the cells with excitation and emmission at 380 nm, 510 nm respectively. Mag-fura-2 acetoxymethyl (AM) dye was obtained from Invitrogen, Massachusetts, USA. ImageJ software (NIH) was used to quantify the fluorescence intensities.

### Immunofluorescence

After fixation, decalcification embedded and sectioned as mentioned above. Sections of mice tibia were stained with NIPA2 and Osterix antibodies (Osx, Q8TDD2, Novus, USA). The sections were incubated with Alexa 488-conjugated goat anti-rabbit (Abcam) and the Alexa 647-conjugated goat anti-mouse (Abcam) as secondary antibodies. Then incubated with 0.1% DAPI for 10 min, NIPA2 and Osx colocalization was evaluated by Image-Pro Plus 6.0. And a mean density value of each visual field was calculated.

Primary osteoblasts were grown in 60 mm dishes and incubated with anti-NIPA2 antibody (1:200) or ALP antibody (1:200) after fixed, permeabilized and blocking with 5% BSA overnight. After stained with 0.1% DAPI, PBS was used to wash and then images were captured. ImageJ software was used to quantify.

Cells were grown in confocal dishes (Nest, California, USA) and incubated with Mitotracker Red CMXRos (M7512, Invitrogen) for 30 min to mark the mitochondria after treatment. Then, the cells were washed with ice-cold PBS and fixed with 4% paraformaldehyde at room temperature for 15 min. Fixed cells were permeabilized with 0.2% Triton X-100 and then blocked with 5% BSA in PBS. After incubation with an antibody against LC3 (1:100 dilution) in the dark at 4 °C for a night, the incubation with a secondary antibody conjugated to Dylight 405 (A0605, Beyotime) sustained for 1 h. The cells were observed and analyzed under a confocal laser scanning microscope (Olympus, Tokyo, Japan). Data were averaged from three independent experiments (n = 90 cells). Image-Pro Plus 6.0 (Media Cybernetics). Analysis of LC3 quantitation and mitochondria colocalization was performed by Image-Pro Plus 6.0 (Media Cybernetics).

### ALP activity detection

The process of ALP activity detection was as previously described in our Meng *et al*.^16^. Briefly, cells were washed with PBS for three times and then lysed by ice-cold 0.1% Triton X-100. Then the lysates were centrifuged at 15000 g for 5 min and the supernatant was collected. Each well of a 96-well plate contained 50 μl supernatant and 150 μl p-nitrophenyl phosphate (Sigma, Irvine, UK) and then incubated for 20 min at 37 °C. Absorbance at 405 nm was detected and total protein concentration was used to normalize the ALP activity.

### ALP staining

ALP was stained by a BCIP/NBT ALP color development kit purchased from Beyotime. Cells were incubated with BCIP/NBT substrate in 60 mm dishes after washed and fixed for 2 hours. Then the images of ALP was collected after washing with PBS.

### Alizarin Red S (ARS) staining

For measurement of bone nodule formation after osteogenic differentiation, detection of the extracellular matrix calcium deposits was determined using ARS staining. Briefly, after culturing with osteogenic medium for 14 days, the cells were washed and fixed routinely, the extracellular matrix calcium deposits were measured by staining the cells with 40 mM alizarin red S solution (Sigma-Aldrich). The cells and nodule formation were captured by phase-contrast microscopy (Nikon, Japan). The density was analyzed by Image-Pro Plus 6.0.

### Lentivirus products

Lentivirus interference experiments was as our previous study^45^. Small interfering RNA (siRNA) sequences targeting human NIPA2 (CATTCATGCTCCAAAGGAA) and Parkin (ACCATTGGGCCTGCTGGTCTA) were used to knock down the expression of NIPA2 and Parkin. The transfection processes were performed by lentiviral vectors respectively expressing NIPA2-RNAi (LV-NIPA2-RNAi), Parkin-RNAi (LV-Parkin-RNAi) and a flag tag. The Lentiviral vectors encoding the amplified NIPA2 sequence (NM_030922, LV-NIPA2) and a flag tag were used to overexpress NIPA2. Lentiviral vectors carrying flag only (LV-Ctrl) were used as a negative control while the nonsilencing siRNA sequence (TTCTCCGAACGTGTCACGT) was used as control. All lentiviral products were designed and synthesized by GeneChem Corporation (GeneChem, Shanghai, China).

### Statistical analysis

The quantitative variables are expressed as the mean ± standard deviation (SD). All data were analyzed using the software program GraphPad Prism 6.02. Statistical significance between two groups was analyzed by Student’s t-test. One-way analysis of variance (ANOVA) was used to compare multiple groups. A P value of <0.05 was considered statistically significant.

### Ethics approval and consent to participate

All the animal experiments were performed in accordance with the National Institutes of Health Guide for the Care and Use of Laboratory Animals and approved by the Institutional Review Board of the First Hospital of China Medical University.

## Data Availability

The datasets used or analyzed during the current study are available from the corresponding author on reasonable request.
